# Association between metabolic score for insulin resistance and prevalence of sarcopenia in US adults: A study based on NHANES 2011 to 2018

**DOI:** 10.1097/MD.0000000000041863

**Published:** 2025-03-14

**Authors:** Hanhui Liu, Yaqi Li, Ye Deng, Zhancheng Liang, Shifeng Feng, Meiqi Fu

**Affiliations:** a Department of Spinal Surgery, Foshan Fosun Chancheng Hospital, Foshan, China.

**Keywords:** insulin resistance, metabolic score for insulin resistance, sarcopenia

## Abstract

This cross-sectional study analyzed National Health and Nutrition Examination Survey data from 2011 to 2018, focusing on individuals aged ≥20 years. The association between metabolic score for insulin resistance (METS-IR) and sarcopenia was examined using weighted multivariable logistic regression, with dose-response relationships characterized by restricted cubic spline analysis. Subgroup and sensitivity analyses were performed, and receiver operating characteristic curve analysis assessed METS-IR’s ability to detect sarcopenia, with the area under the curve used for evaluation. The study included 4553 participants (mean age, 40 years; 49.4% male and 50.6% female). In the descriptive analysis, METS-IR levels in sarcopenia (mean, 52.39) were significantly higher than METS-IR levels in nonsarcopenia (mean, 41.94), indicating an association with sarcopenia. A univariate logistic regression analysis showed that sarcopenia and METS-IR were positively correlated. Even after accounting for all variables, METS-IR maintained a stable positive correlation with the prevalence of sarcopenia (odds ratio, 1.06 [95% CI, 1.06–1.08]). The results remained stable when METS-IR was categorized into quartiles. METS-IR was found to positively correlate with sarcopenia prevalence using restricted cubic spline analysis. According to subgroup analysis, there is a consistent and stable positive correlation between the prevalence of sarcopenia and METS-IR. Sensitivity analysis showed that METS-IR and sarcopenia continued to have a significant positive connection even after excluding extreme findings. The area under the curve value of METS-IR in the receiver operating characteristic curve analysis was 0.7217, suggesting that METS-IR could be a useful predictor of sarcopenia.

## 1. Introduction

A systemic skeletal muscle illness called sarcopenia that worsens with age is marked by a loss of muscular mass, strength, and function.^[1]^ Sarcopenia has received increasing attention in recent years due to its association with various adverse health outcomes, including falls, functional decline, frailty, hospitalization, and increased mortality.^[2]^ Over the past few decades, the European Working Group on Sarcopenia in Older People has periodically improved the diagnostic and criteria for sarcopenia, drawing more attention from medical experts.^[3]^ Systematic reviews of studies in the general population report a global prevalence of sarcopenia of ≈10% in men and women.^[4]^ Because sarcopenia has a major negative influence on both healthcare expenses and patients’ quality of life,^[5]^ developing efficient preventative measures is crucial.

A physiological condition known as insulin resistance (IR) is typified by diminished target tissue responsiveness to physiological insulin levels and impaired efficacy of insulin signal transmission.^[6]^ In skeletal muscle, the liver, and adipose tissue, it is characterized by decreased insulin sensitivity.^[7]^ An essential part of the metabolic syndrome and a key player in its pathogenesis are IRs.^[8,9]^ Even though the glucose clamp method is presently the “gold standard” for evaluating IR, large-scale epidemiological research should not use it because of its invasiveness, complexity, and high expense.^[10]^ Indirectly assessing IR without requiring fasting insulin testing is the metabolic score for IR (METS-IR), which was originally described in 2018. It takes into account blood glucose, triglycerides (TGs), body mass index (BMI), and high-density lipoprotein-cholesterol (HDL-C).^[11]^ Insulin-induced glucose metabolism mostly occurs in skeletal muscle, and IR and metabolic syndrome are strongly associated with muscle mass loss.^[12]^ Studies on the pathogenesis of sarcopenia have reported that muscle cells affected by IR, local hyperlipidemia, and systemic inflammation might exacerbate the development of sarcopenia.^[13]^ Moreover, several research investigations have shown a direct correlation between the METS-IR score and a variety of medical conditions, including hyperuricemia, diabetes, and heart disease.^[14–16]^ However, no previous research has examined the relationship between the METS-IR score and sarcopenia. One useful prediction indicator that may be used to forecast when sarcopenia will develop is the METS-IR index. It is simple to use, reliable, and repeatable. Using the METS-IR index clinically to predict sarcopenia may help with early intervention and health advice for high-risk individuals, potentially lowering the risk of sarcopenia and providing information for more in-depth study. Given the high prevalence of IR in various populations, we hypothesize that elevated METS-IR scores could serve as an early indicator of sarcopenia risk, enabling better preventative strategies for high-risk individuals.

## 2. Materials and methods

### 2.1. Description of the survey and study population

A complicated, stratified, multistage sample method is used in the cross-sectional National Health and Nutrition Examination Survey (NHANES) to evaluate the nutritional status and general health of the American people. The trial was authorized by the ethical review board, and each participant provided written consent. This research made use of 4 cycles’ worth of data from the NHANES 2011 to 2018. The inclusion criteria were participants aged >20 years, participants with complete sarcopenia data, and participants with complete METS-IR data.

### 2.2. Calculation of METS-IR

The subjects’ blood was drawn in the morning to assess their fasting TG and glucose levels following an 8.5-hour overnight fast. Enzymatic techniques were employed to ascertain these levels, which were then quantified by an automated biochemical analyzer. The following formula can be used to calculate METS-IR, per earlier research: METS-IR = Ln(TGs [mg/dL] + 2 × blood glucose [mg/dL])×BMI/Ln HDL-C (mg/dL).^[11]^ By using magnesium sulfate/dextran to block non-HDL-C and polyethylene glycol-modified enzymes to convert HDL-C into a detectable chromophore, a specific measurement of HDL-C is achieved. All laboratory indicators and measurements can be found in the NHANES Laboratory Quality Control Report (https://wwwn.cdc.gov/nchs/nhanes/default.aspx).

### 2.3. Definition of sarcopenia

NHANES measured the appendicular skeletal muscle mass, which comprises nonfat and nonbone tissue, using dual-energy X-ray absorptiometry. The ratio of total appendicular skeletal muscle mass (kg) to BMI (kg/m^2^) yields the sarcopenia index. BMI is measured by a trained professional on an minimum effective concentration mobile screening cart. According to the standards of the Foundation for the National Institutes of Health Biomarkers Collaboration Sarcopenia Project, a sarcopenia index of <0.789 for men and 0.512 for women is considered sarcopenic. These criteria have been verified in prior research.^[17]^

### 2.4. Covariates

This study included a range of covariates, encompassing demographic characteristics, lifestyle factors, and health status. Demographic characteristics included age, sex, race, poverty index ratio, and education level. Lifestyle variables encompassed smoking behavior and physical activity level. A questionnaire was used to assess smoking behavior; people who had smoked >100 cigarettes in their lives were considered smokers. The Global Physical Activity Questionnaire was utilized to evaluate the degree of physical activity, and metabolic equivalent (MET) values were calculated using the formula: MET (minutes/week) = MET × frequency per week × duration per activity.^[18]^ Physical inactivity was defined as having MET values <600 minutes per week. Health status, including diabetes, hypertension, hypercholesterolemia, and chronic renal disease, was assessed based on self-reports or diagnoses from medical professionals. Based on self-reported histories of angina, heart failure, and coronary heart disease, the status of coronary heart disease was established.

### 2.5. Statistical methods

Data from 2011 to 2018 were collected from the NHANES database, encompassing 4 survey cycles. Missing values for the exposure and outcome variables were addressed by excluding samples with incomplete data, whereas missing values for covariates were handled using the omission method. The baseline features of the final individuals were grouped by sarcopenia status using descriptive analysis. Logistic regression analysis was employed to investigate the relationship between METS-IR and sarcopenia, adjusting for various covariates. The continuous variable METS-IR was converted into quartiles (Q1: <33.54; Q2: 33.54–41.13; Q3: 41.13–49.92; and Q4: >49.92) in order to examine the association between different METS-IR levels and sarcopenia. The nonlinear relationship was evaluated using a 3-node restricted cubic spline (RCS) model. Node selection was performed using cross-validation, and the presence of nonlinear effects was assessed with the likelihood ratio test. Sensitivity and subgroup analyses were carried out to assess the robustness and consistency of the findings. The effectiveness of METS-IR to predict sarcopenia was lastly assessed using receiver operating characteristic (ROC) curve analysis. R software (version 4.2.3) was used for all analyses, and *P* < .05 was used as the statistical significance criterion.

## 3. Results

### 3.1. Characteristics of the studied population

**Figure 1.**
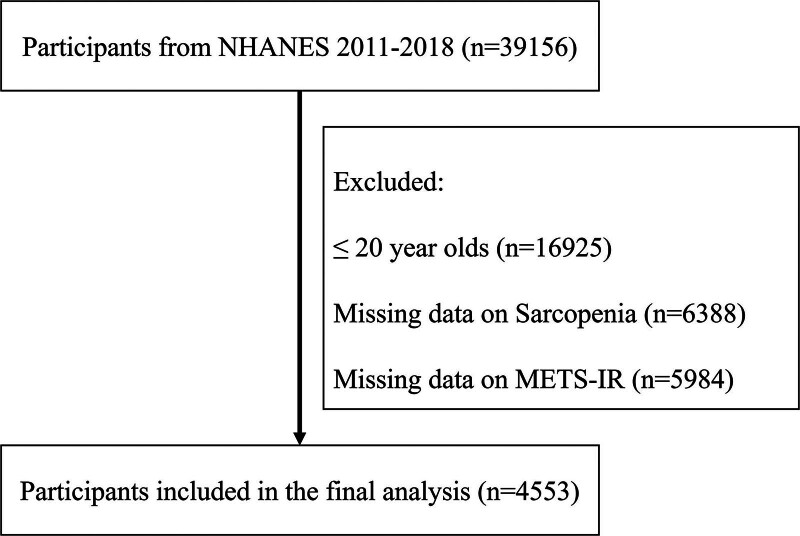
Include participants in the process. METS-IR = metabolic score for insulin resistance, NHANES = National Health and Nutrition Examination Survey.

Data extracted from the NHANES database, as shown in Figure 1, encompassed a total of 4553 participants, with 4130 nonsarcopenic and 423 sarcopenic individuals. As shown in Table 1, participant characteristics were classified according to whether or not they had sarcopenia. Compared to those without sarcopenia, sarcopenic patients were generally older, predominantly of Mexican ethnicity, and had lower educational levels and income. In addition, sarcopenic patients were more likely to have diabetes, cardiovascular diseases, chronic kidney disease, hypertension, and hypercholesterolemia. Notably, these patients exhibited higher METS-IR levels, indicating a correlation with sarcopenia.

**Table 1:** Baseline characteristics of the study population.

Characteristic	Level	Overall	Nonsarcopenia	Sarcopenia	*P* value
N		4553	4130	423	
Age, yr (%)	<40	2326 (51.1)	2184 (52.9)	142 (33.6)	<.001
	>40	2227 (48.9)	1946 (47.1)	281 (66.4)	
Sex, %	Female	2304 (50.6)	2092 (50.7)	212 (50.1)	.874
	Male	2249 (49.4)	2038 (49.3)	211 (49.9)	
Race, %	Mexican-American	697 (15.3)	555 (13.4)	142 (33.6)	<.001
	Non-Hispanic Black	881 (19.3)	858 (20.8)	23 (5.4)	
	Non-Hispanic White	1597 (35.1)	1480 (35.8)	117 (27.7)	
	Others	1378 (30.3)	1237 (30.0)	141 (33.3)	
Education level, %	Above high school	2733 (60.0)	2554 (61.8)	179 (42.3)	<.001
	High school	964 (21.2)	869 (21.0)	95 (22.5)	
	Under high school	856 (18.8)	707 (17.1)	149 (35.2)	
PIR, %	<1	909 (21.8)	800 (21.1)	109 (28.8)	<.001
	1–3	1700 (40.8)	1535 (40.5)	165 (43.5)	
	>3	1557 (37.4)	1452 (38.3)	105 (27.7)	
Activity status, %	Active	2791 (61.3)	2586 (62.6)	205 (48.5)	<.001
	Inactive	1762 (38.7)	1544 (37.4)	218 (51.5)	
Smoke, %	No	2730 (60.0)	2477 (60.0)	253 (59.8)	.900
	Yes	1821 (40.0)	1651 (40.0)	170 (40.2)	
	No record	2 (0.0)	2 (0.0)	0 (0.0)	
Diabetes, %	No	4113 (90.3)	3768 (91.2)	345 (81.6)	<.001
	Yes	349 (7.7)	286 (6.9)	63 (14.9)	
	No record	91 (2.0)	76 (1.8)	15 (3.5)	
CAD, %	No	4441 (97.5)	4037 (97.7)	404 (95.5)	.008
	Yes	112 (2.5)	93 (2.3)	19 (4.5)	
CKD, %	No	4458 (97.9)	4056 (98.2)	402 (95.0)	<.001
	Yes	93 (2.0)	72 (1.7)	21 (5.0)	
	No record	2 (0.0)	2 (0.0)	0 (0.0)	
Hypertension, %	No	3444 (75.6)	3166 (76.7)	278 (65.7)	<.001
	Yes	1102 (24.2)	959 (23.2)	143 (33.8)	
	No record	7 (0.2)	5 (0.1)	2 (0.5)	
Hypercholesterolemia, %	No	3384 (74.3)	3118 (75.5)	266 (62.9)	<.001
	Yes	1156 (25.4)	1003 (24.3)	153 (36.2)	
	No record	13 (0.3)	9 (0.2)	4 (0.9)	
BMI, kg/m^2^; mean (SD)		28.84 (6.84)	28.34 (6.54)	33.73 (7.69)	<.001
HDL, mg/dL; mean (SD)		52.80 (15.14)	53.21 (15.21)	48.74 (13.84)	<.001
TG, mg/dL; mean (SD)		120.31 (122.21)	116.70 (120.50)	155.51 (132.95)	<.001
GLU, mg/dL; mean (SD)		106.48 (34.93)	105.18 (32.63)	119.16 (50.70)	<.001
METS-IR, mean (SD)		42.91 (12.60)	41.94 (12.00)	52.39 (14.33)	<.001
ASM, kg; mean (SD)		22.64 (6.36)	22.87 (6.33)	20.37 (6.17)	<.001
SMI, mean (SD)		0.80 (0.20)	0.82 (0.20)	0.61 (0.14)	<.001

Mean (SD) for continuous variables and % for categorical variables.

ASM = appendicular skeletal muscle, BMI = body mass index, CAD = coronary artery disease, CKD = chronic kidney disease, GLU = glucose, HDL = high-density lipoprotein, METS-IR = metabolic score for insulin resistance, PIR = poverty index ratio, SMI = skeletal muscle index, TG = triglyceride.

### 3.2. Association between METS-IR and sarcopenia

**Figure 2.**
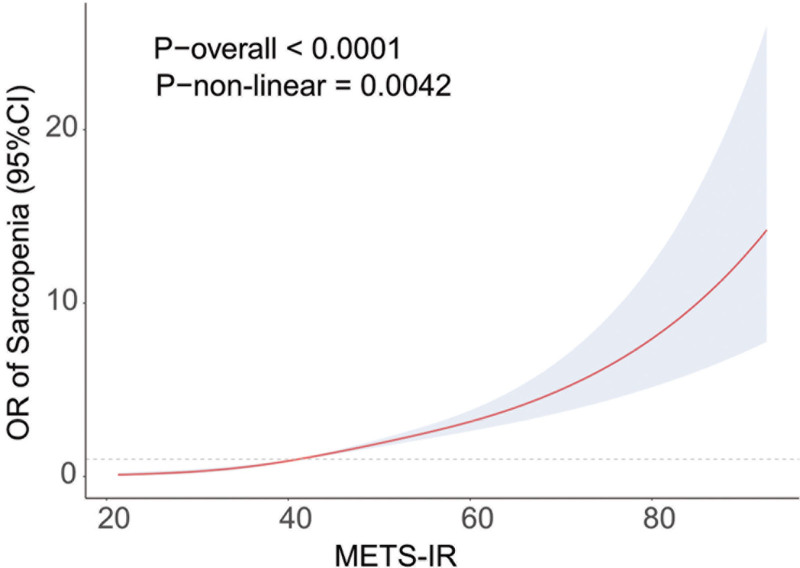
Restricted cubic spline (RCS) curve fits the association of metabolic score for insulin resistance (METS-IR) with sarcopenia.

This study employed logistic regression analysis to assess the relationship between METS-IR and the prevalence of sarcopenia, as shown in Table 2. In the unadjusted model 1, METS-IR was significantly associated with an increased prevalence of sarcopenia (odds ratio [OR], 1.06 [95% CI, 1.05–1.07]). The fully adjusted model 3 showed that when multiple factors were included one by one, METS-IR remained stably positively correlated with the prevalence of sarcopenia (OR, 1.06 [95% CI, 1.06–1.08]). METS-IR was classified as a categorical variable in order to assess the relationship between METS-IR levels and sarcopenia in more detail. Even after correcting for all variables, there was a significant correlation between the highest quartile of METS-IR and the lowest quartile (OR, 13.6 [95% CI, 8.40–21.90]). The RCS analysis is shown in Figure 2, which shows a strong positive association between METS-IR and sarcopenia. It also shows that elevated METS-IR levels are substantially linked to an increased prevalence of sarcopenia.

**Table 2:** The relationship between METS-IR and sarcopenia.

		Model 1: OR (95% CI); *P* value	Model 2: OR (95% CI); *P* value	Model 3: OR (95% CI); *P* value
Sarcopenia	METS-IR	1.06 (1.05–1.07); <.001	1.07 (1.06–1.08); <.001	1.06 (1.05–1.07); <.001
	Q1	Reference	Reference	Reference
	Q2	3.25 (1.85–5.68); <.001	2.99 (1.68–5.30); <.001	3.20 (1.87–5.48); <.001
	Q3	5.64 (3.39–9.37); <.001	4.76 (2.80–8.12); <.001	5.10 (2.95–8.84); <.001
	Q4	14.60 (8.68–24.60); <.001	13.80 (8.07–23.60); <.001	13.60 (8.40–21.90); <.001
	*P*_trend_	<.001	<.001	<.001

Model 1: no covariates adjusted. Model 2: adjusted for age, sex, and race. Model 3: adjusted for age, sex, race, educational level, poverty index ratio, smoke, activity status, hypertension, hypercholesterolemia, coronary artery disease, chronic kidney disease, and diabetes.

METS-IR = metabolic score for insulin resistance, OR = odds ratio.

### 3.3. Subgroup analysis and sensitivity analysis

The association between METS-IR and sarcopenia prevalence across stratification criteria was examined using subgroup analysis. In all categories, there was a consistent positive correlation between METS-IR and the prevalence of sarcopenia, as shown in Table 3. Interaction tests revealed a significant interaction effect with gender. In addition, after excluding extreme METS-IR values (±3 SD) and diabetic patients, sensitivity analysis was performed on the remaining 4087 participants. METS-IR and sarcopenia prevalence are positively correlated, and the results of the sensitivity analysis corroborated the study’s main conclusions, as shown in Table, Supplement Digital Content, http://links.lww.com/MD/O528.

**Table 3:** Subgroup analysis between METS-IR and sarcopenia.

Characteristic	Group	OR (95% CI); *P* value	*P*_interaction_
Age, yr	<50	1.06 (1.05–1.08); <.001	.800
	>50	1.07 (1.05–1.08); <.001	
Sex	Male	1.08 (1.07–1.10); <.001	<.001
	Female	1.05 (1.03–1.06); <.001	
Activity status	Active	1.07 (1.05–1.08); <.001	.600
	Inactive	1.06 (1.05–1.07); <.001	
Smoke	No	1.06 (1.05–1.07); <.001	.700
	Yes	1.07 (1.05–1.09); <.001	
Hypertension	No	1.06 (1.05–1.08); <.001	.900
	Yes	1.07 (1.05–1.09); <.001	
Hypercholesterolemia	No	1.06 (1.05–1.08); <.001	.700
	Yes	1.07 (1.05–1.09); <.001	
Diabetes	No	1.07 (1.06–1.08); <.001	.200
	Yes	1.05 (1.02–1.08); <.001	

METS-IR = metabolic score for insulin resistance, OR = odds ratio.

### 3.4. ROC analysis

**Figure 3.**
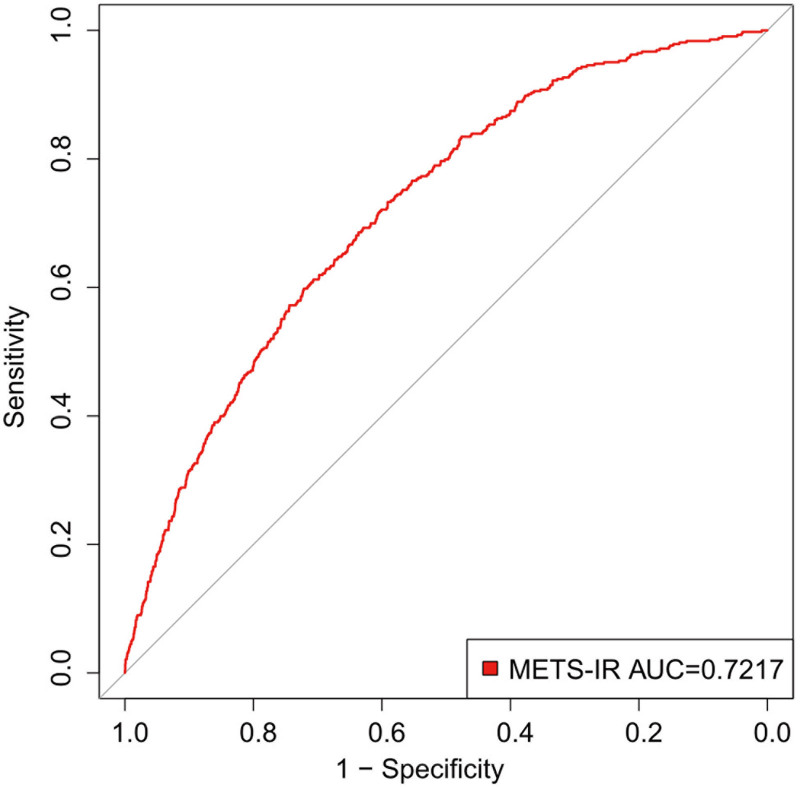
Receiver operating characteristic (ROC) curves for metabolic score for insulin resistance (METS-IR) prediction of sarcopenia. AUC = area under the curve.

The area under the curve (AUC) value and the ROC curve for the prediction of sarcopenia are shown in Figure 3. The findings show that METS-IR has an AUC value of 0.7217, indicating that it may be a useful predictor of sarcopenia for early screening and the creation of preventative interventions.

## 4. Discussion

This cross-sectional study included 4553 participants with the aim of examining the relationship between METS-IR and sarcopenia prevalence. The findings showed that the frequency of sarcopenia and METS-IR had a substantial positive association. In addition, when METS-IR was converted from a continuous to a categorical variable using quartiles, the trend persisted. A nonlinear positive connection between METS-IR and the frequency of sarcopenia was found using RCS analysis. Moreover, sensitivity analysis and subgroup analysis verified the strength of the positive correlation between METS-IR and sarcopenia prevalence. According to ROC analysis, METS-IR may be able to predict the onset of sarcopenia.

A prevalent ailment among the elderly is sarcopenia, which is typified by a reduction in muscle mass, quality, and function.^[12]^ Previous cross-sectional studies have primarily focused on the relationship between blood biomarkers or anthropometric indicators (such as BMI, body roundness index, and weight-adjusted waist index) and sarcopenia.^[19–21]^ This study provides evidence linking comprehensive metabolic indicators to muscle health. Sarcopenia prevalence and METS-IR were shown to be positively correlated in this research. By incorporating various metabolic parameters, METS-IR offers a more comprehensive assessment of the metabolic factors affecting sarcopenia. Because insulin-mediated glucose metabolism mostly occurs in skeletal muscle, muscle mass is thought to be associated with IR.^[22]^ Many reports suggest a negative correlation between IR and muscle mass, which may lead to an increased risk of sarcopenia.^[23,24]^ In a cross-sectional research study with 14,528 subjects, sarcopenia was associated with poor glucose metabolism, with the association most pronounced in individuals under 60 years old.^[25]^ A 6-year Korean study of elderly patients with diabetes used computed tomography to measure mid-thigh muscle cross-sectional area and oral glucose tolerance tests, concluding that type 2 diabetes is associated with muscle loss and that IR accelerates skeletal muscle protein degradation.^[26]^ Similarly, descriptive research comprising 4030 patients employed the TG glucose (TyG) index as a measure of IR. Sarcopenia and the TyG index were observed to positively correlate (OR, 1.31 [95% CI, 1.07–1.60]) for every unit rise in the TyG index, highlighting the critical role that IR plays in sarcopenia.^[27]^ Based on the ROC analysis (AUC, 0.7217), this study suggests that METS-IR is a potential predictive marker for sarcopenia, offering value in screening and early assessment.

Various potential mechanisms contribute to the development of sarcopenia, but the underlying mechanisms linking METS-IR and sarcopenia have not been fully elucidated. Sarcopenia is an inflammatory condition driven by proinflammatory cytokines and oxidative stress.^[28]^ Numerous disorders associated with muscle loss have been related to IR, oxidative stress, inflammatory cytokines, and physical inactivity.^[29]^ IR is closely related to inflammation and may be a key factor in muscle mass loss, as IR suppresses insulin’s anti-inflammatory effects, thereby potentially promoting inflammation.^[30–32]^ The mechanisms through which insulin reduces inflammation have been studied. These mechanisms include suppression of the formation of monocyte chemoattractant protein and plasma soluble intercellular adhesion molecule, reduction of p47phox and reactive oxygen species in monocytes, and upregulation of inhibitor of NF-κB and downregulation of nuclear factor kappa B in monocytes.^[33]^ In addition, compensatory hyperinsulinemia caused by IR adversely affects glycogen synthesis, accelerates protein degradation, and reduces protein synthesis.^[34]^ In addition, myostatin levels rise as a result of IR-induced hyperinsulinemia, which lowers bone mass.^[35]^ Lipids also contribute to the development of sarcopenia by impairing lipid signaling pathways mediated by adipose TG lipase and peroxisome proliferator-activated receptor α in adipocytes, leading to the upregulation of proinflammatory cytokines.^[36]^ Furthermore, TG accumulation in muscle cells involves various pathways, such as muscle fat degeneration related to intramyocellular lipid through fibrous/fatty progenitor cells, and ceramide accumulation, which also has a deleterious effect on skeletal muscle function.^[37]^ The biochemical processes underlying the association between sarcopenia and the new indicator METS-IR, which combines glucose, TG, HDL-C, and BMI, need to be further explored.

There are several advantages to our study. The main benefit is the innovative application of METS-IR to examine its association with the risk of sarcopenia, providing new perspectives on the connection between IR and sarcopenia. Second, the NHANES database has a large sample size allowing for robust statistical analyses that reduce variability in effect size estimates. Furthermore, our investigation revealed a persistent positive correlation between the incidence of sarcopenia and METS-IR, indicating a noncoincidental association. METS-IR is a readily available marker that can be computed in clinical practice using standard blood parameters, offering insightful information for sarcopenia screening, prevention, and therapy in primary healthcare settings. However, our study also has several limitations. First, as a cross-sectional study, it cannot establish a causal relationship between METS-IR and sarcopenia. Second, any confounding factors that may have influenced the results were excluded from the study, despite the fact that it accounted for a number of covariates to examine the relationship between METS-IR and sarcopenia. In this study, various indicators for assessing IR were not compared using ROC curves. The METS-IR score was selected as the primary assessment tool due to its widespread recognition and robust predictive performance. While other indicators may contribute to the findings, the absence of comparison does not alter the conclusions. Finally, certain potentially influential factors (eg, diet and medication status) were not taken into account, and future studies may further investigate their impact on METS-IR.

## 5. Conclusion

A new assessment for the prevention of sarcopenia, the METS-IR, has been found to be positively associated with the prevalence of sarcopenia in US adults.

## Acknowledgments

The authors are appreciative that the National Center for Medical Research at the Institute of Prevention and Control of Disorders has made the National Health and Nutritional Evaluation Survey available to all citizens of the country.

## Author contributions

**Data curation:** Hanhui Liu, Yaqi Li.

**Formal analysis:** Hanhui Liu, Ye Deng.

**Investigation:** Hanhui Liu, Zhancheng Liang.

**Methodology:** Hanhui Liu, Shifeng Feng, Meiqi Fu.

**Project administration:** Hanhui Liu, Yaqi Li, Meiqi Fu.

**Supervision:** Hanhui Liu, Yaqi Li, Meiqi Fu.

**Writing – original draft:** Hanhui Liu, Meiqi Fu.

**Writing – review & editing:** Hanhui Liu, Meiqi Fu.

**Conceptualization:** Meiqi Fu.

## Supplementary Material


