# Supplementary material for: Association between metabolic score for insulin resistance and prevalence of sarcopenia in US adults: A study based on NHANES 2011 to 2018
Source: Medicine (Baltimore). 2025 Mar 14;104(11):e41863. doi: 10.1097/MD.0000000000041863 (PMC11922397; doi:10.1097/MD.0000000000041863)
Supplement: Supplementary file 1 [file medi-104-e41863-s001.docx]

**Supplementary Table 1 Sensitivity analysis between METS_IR and Sarcopenia.**

|  |  | Model 1  OR (95%CI) P-value | Model 2  OR (95%CI) P-value | Model 3  OR (95%CI) P-value |
| --- | --- | --- | --- | --- |
| Sarcopenia | METS-IR | 1.07 (1.06, 1.09) <0.001 | 1.08 (1.06, 1.09) <0.001 | 1.07 (1.06, 1.09) <0.001 |
|  | Q1 | [Reference] | [Reference] | [Reference] |
|  | Q2 | 4.67 (2.67, 8.15) <0.001 | 4.40 (2.54, 7.63) <0.001 | 4.22 (2.41, 7.38) <0.001 |
|  | Q3 | 8.34 (5.17, 13.5) <0.001 | 7.25 (4.43, 11.9) <0.001 | 7.19 (4.17, 12.4) <0.001 |
|  | Q4 | 19.2 (11.7, 31.8) <0.001 | 18.7 (11.6, 30.2) <0.001 | 16.8 (10.2, 27.8) <0.001 |
|  | P for trend | <0.001 | <0.001 | <0.001 |

CI: Confidence Interval; METS_IR: Metabolic score for insulin resistance; OR: Odds Ratio;

Model 1: No covariates adjusted; Model 2: Adjusted for Age, Sex, and Race; Model 3: Adjusted for age, Sex, Race, Educational level, PIR, Smoke, Activity status, Hypertension, Hypercholesterolemia, CAD, CKD, Diabetes.
